# Bone Metastasis and the Immunosuppressive Tumor Microenvironment: Mechanisms and Therapeutic Strategies

**DOI:** 10.32604/or.2026.090531

**Published:** 2026-10-28

**Authors:** Minghao Ba, Yuzhu Chen, Peng Jiang, Shanshan Cai, Xin Huang, Tongmei Zhang

**Affiliations:** 1 Affiliated School of Pediatrics, Nanjing Medical University, Nanjing, China; 2 Department of Oncology, Beijing Chest Hospital, Capital Medical University, Beijing Tuberculosis and Thoracic Tumor Research Institute, Beijing, China; 3 Division of Biomedical and Life Sciences, Faculty of Health and Medicine, Lancaster University, Lancaster, UK; 4 College of Health Science and Technology, Shanghai Jiao Tong University School of Medicine, Shanghai, China

**Keywords:** Bone metastasis, immunosuppressive microenvironment, tumor-associated macrophages, T-cell exhaustion, vicious cycle, immune checkpoint inhibitors, metabolic reprogramming, extracellular vesicles

## Abstract

Numerous solid tumours have bone as one of their most common sites of distant metastasis, and bone metastasis causes skeletal-related events (SREs) that impair quality of life and portend poor prognosis. Bone metastasis arises not from invasive tumour cells alone but through mutual adaptation and co-evolution between tumour cells and the bone microenvironment. A key mechanism of immune evasion is the establishment of an immunosuppressive microenvironment, wherein tumour-derived cytokines, metabolic reprogramming, and extracellular vesicles drive M2 macrophage polarization, CD8^+^ T-cell exhaustion, regulatory T-cell enrichment, and natural killer cell dysfunction. Transforming growth factor-β (TGF-β) released during the “tumour-bone” vicious cycle further amplifies local immunosuppression, and metabolic reprogramming depletes glucose and amino acids while accumulating lactate, directly impairing effector T-cell function. Immune checkpoint inhibitor (ICI) therapy shows limited efficacy in bone metastases; however, combination strategies including bone-protective agents (denosumab, bisphosphonates), metabolic modulators, cytokine- or extracellular vesicle-targeted agents, novel nanodelivery systems, and engineered cell therapies demonstrate encouraging preclinical and early clinical promise for reversing immunosuppression. Translation still faces major challenges, including microenvironmental heterogeneity, a lack of bone-specific biomarkers, combination-related toxicity, and delivery bottlenecks. This review aims to synthesize the mechanisms underlying the immunosuppressive bone-metastasis microenvironment and to appraise current and emerging combination strategies, thereby informing individualized treatment for patients with bone metastases.

## 1 Introduction

Bone is one of the most common sites of distant metastasis for various solid tumors, with breast cancer, prostate cancer, and lung cancer having the highest rates of bone metastasis. An analysis of large-scale CT reports using natural language processing technology shows that the five-year cumulative incidence of bone metastasis for prostate cancer, breast cancer, head and neck cancer, and lung cancer is as high as 52%, 41%, 36%, and 33%, respectively [1]. These estimates are derived from CT reports at a single tertiary cancer center and reflect imaging-detected incidence in a selected referral population rather than population-level incidence. Population-based estimates may differ. Bone metastases can not only lead to SREs—such as pathological fractures, spinal cord compression, and intractable bone pain—which severely impair patients’ quality of life, but can also significantly shorten overall survival [2]. Therefore, in-depth analysis of the pathological mechanisms underlying bone metastases and the development of effective treatment strategies have become critical areas requiring urgent breakthroughs in the field of oncology. This review focuses specifically on bone metastases arising from solid carcinomas, with particular emphasis on breast, prostate, and lung cancers. Primary malignant bone tumors (such as osteosarcoma and Ewing sarcoma) are not within the scope of this review, as their biological behavior, microenvironment composition, and clinical management differ substantially from carcinoma bone metastases. Where evidence from other disease models is discussed due to the paucity of bone metastasis-specific data, this is explicitly indicated.

Ultimately, the formation and development of bone metastasis is a complex process of mutual adaptation and synergistic evolution of the tumor cells and the different elements of the bone microenvironment. The hematopoietic bone marrow, where immune cells develop, is the first place where tumor cells “hijack” the microenvironment, turning this organ, which normally provides immune surveillance, into a “sanctuary” that allows the tumor to escape the immune system [2]. Once the tumour cells have invaded the bone, they can alter bone homeostasis in many ways: They stimulate bone resorption by the osteoclast, but also inhibit bone formation by the osteoblast. At the same time, growth factors like transforming growth factor-β (TGF-β) released from the bone matrix then stimulate the proliferation of tumor cells, creating a “vicious cycle” typical of bone metastasis [3]. Significantly, the few cytokines involved in this cycle, in particular, TGF-β, are instrumental in tumor progression and also dramatically alter the bone microenvironment by attracting regulatory T cells (Tregs) and myeloid-derived suppressor cells (MDSCs) and by inhibiting effector T cell function [3]. The rapidly evolving landscape of single-cell sequencing and spatial omics technologies has significantly expanded our knowledge of the bone metastasis immune ecosystem in recent years. Researchers have identified microenvironment profiles characterized by a dominance of myeloid cells and a high enrichment of immunosuppressive signals in various bone metastasis tissues, including those from prostate, breast, and lung cancers [2].

In the immunosuppressive microenvironment of bone metastasis, multiple immune cell subsets undergo profound functional remodeling. MDSCs are key immunosuppressive effector cells in bone metastasis; they suppress the function of T cells and natural killer (NK) cells and further differentiate into tumor-associated macrophages (TAMs), monocytes, and dendritic cells (DCs), thereby comprehensively promoting the occurrence and progression of bone metastasis. TAMs exhibit a pronounced M2-type polarization bias and further exacerbate the immunosuppressive state of the microenvironment by secreting immunosuppressive factors such as IL-10 and TGF-β. T-cell functional exhaustion and the local accumulation of Tregs are also key mechanisms by which bone metastases achieve immune evasion [4]. Furthermore, tumor-derived extracellular vesicles (EVs), which carry bioactive molecules such as miRNAs, proteins, and lipids, play a critical role in long-range signaling during the shaping of the pre-metastatic bone microenvironment, the regulation of immune cell function, and tumor-bone cell interactions. Their mechanisms—including the expansion of MDSCs, the impairment of lymphocyte function, and the regulation of the osteogenesis/resorption balance—collectively contribute to the establishment of the bone metastasis niche [5]. Metabolic reprogramming, as a core survival strategy for tumor cells to adapt to the bone microenvironment, not only provides energy and substrates for biosynthesis but also leads to lactic acid accumulation and microenvironmental acidification, which directly suppress effector T-cell function, thereby forming a mutually reinforcing “metabolic-immune-bone” pathological network [6].

With the emergence of immune checkpoint inhibitors (ICIs), the treatment landscape of various types of advanced solid tumors has changed dramatically, but the results in patients with bone metastases have not been what was hoped for. In clinical trials comprising of multiple centers, patients with metastases in bone had a significantly shorter overall survival after ICI, and the presence of bone metastases was an independent predictor of poor prognosis with ICI [2]. Mechanistic studies have shown that the osteoclasts from bone metastases, under the influence of tumor cell domestication, produce a lot of osteopontin (OPN). OPN is carried by the blood to extraosseous sites, and there it has a remote inhibitory effect on the systemic efficacy of immunotherapy, through inhibition of recruitment and differentiation of important T-cell subsets, including the CD8^+^TCF1^+^ precursor exhaustion T cells (Tpex) [7]. This discovery not only helps to explain why bone is considered an “immunoregulatory organ” in systemic immune homeostasis but also indicates that targeting the tumor cells *per se* may not be enough to break immune resistance caused by the bone metastases.

Because the creation of the immunosuppressive microenvironment of the bone metastases is a complex process involving multiple cell types, signaling pathways, and regulatory levels, single-target therapeutic approaches do not necessarily yield the best therapeutic effect. At present, clinical trials combining ICIs with bone protection therapies (e.g., bisphosphonates or denosumab) have shown some preliminary promise of synergy, but the potential for optimally combining arming the immune system against the tumor while protecting the bone has yet to be systematically investigated [8]. In the meantime, novel approaches like targeting cytokine/EV pathways, combination of metabolic modulators and immunotherapy, and new biomaterials are slowly taking the spotlight for research [6]. But the challenge is still in the future to effectively translate these basic research findings into clinically accessible treatment regimens.

Specifically, this article aims to provide a comprehensive overview of the mechanisms underlying the development of the immunosuppressive microenvironment in bone metastases from four perspectives: how tumor cells manipulate the function of immune cells; immune regulatory mechanisms in the “vicious cycle”; how signaling networks are relayed by cytokines and EVs; and how metabolic reprogramming influences the microenvironment. Based on this, the paper summarizes existing targeted intervention strategies based on immunotherapy and their clinical translation development process, offering a theoretical basis and reference framework for precision treatment of bone metastasis.

## 2 Review Methods

This is a narrative review that synthesizes the current understanding of the immunosuppressive microenvironment in bone metastases and emerging therapeutic strategies. Literature retrieval was performed in PubMed, with the final search date set to April 2026. The full search string was: (“bone metastasis”) AND (“immunosuppressive microenvironment” OR “tumor-associated macrophages” OR “T cell exhaustion” OR “vicious cycle” OR “immune checkpoint inhibitors” OR “metabolic reprogramming” OR “extracellular vesicles”).

For study eligibility, we included original research articles, comprehensive review articles, and published clinical trial reports offering representative evidence related to the bone-metastatic immunosuppressive microenvironment and relevant therapeutic approaches. Exclusion criteria comprised case reports, conference abstracts without full-text publication, and studies irrelevant to the scope of this review. We manually screened titles, abstracts, and full-text content for final literature selection.

## 3 Mechanisms Underlying the Formation of an Immunosuppressive Microenvironment in Bone Metastases

Tumor cell-mediated immune remodeling, cytokine/exosome signaling networks (Fig. 1), the bone-tumor vicious cycle, and metabolic reprogramming (Fig. 2) act synergistically to create a highly immunosuppressive microenvironment in bone metastases.

**Figure 1:**
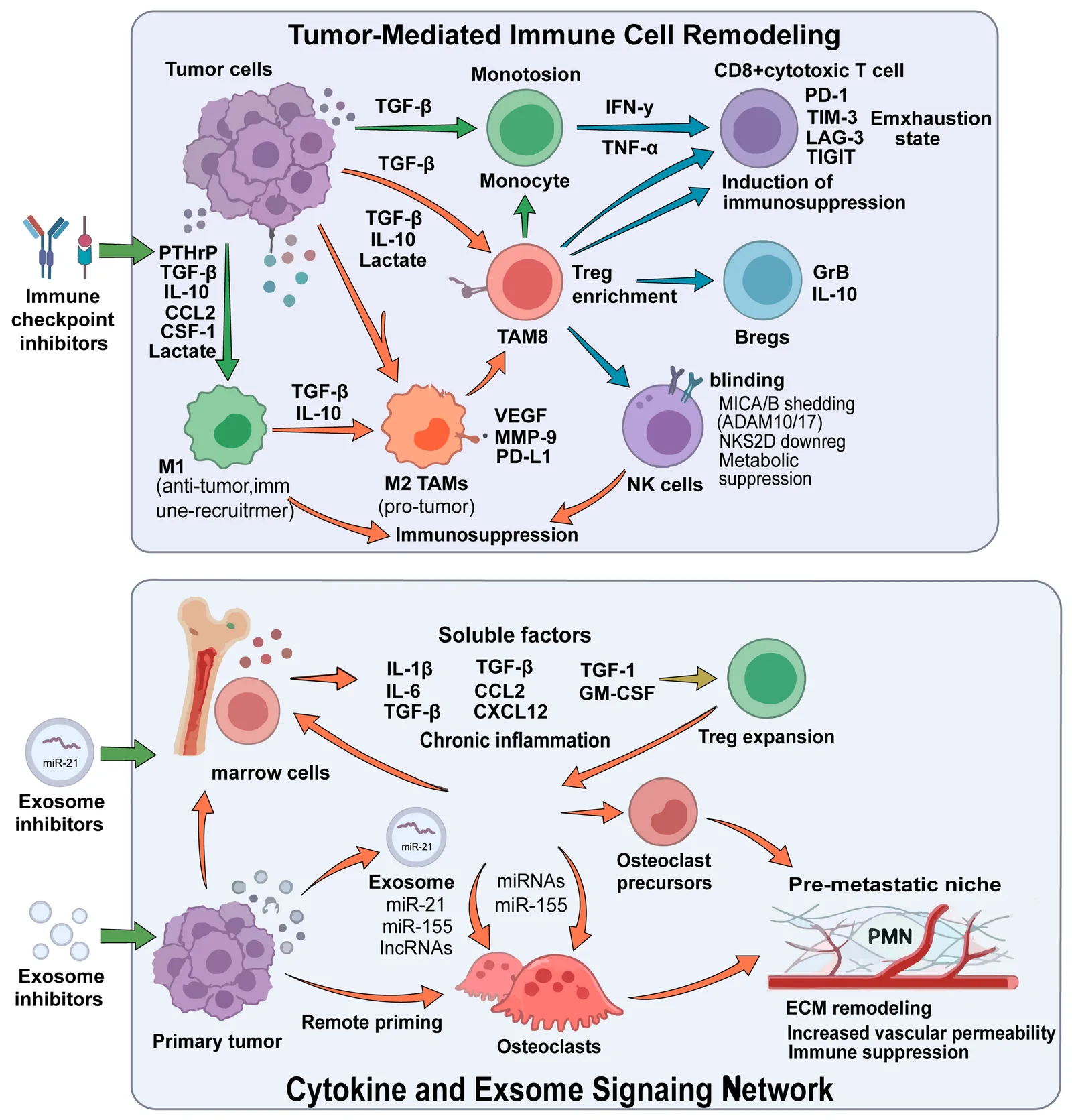
Tumor-mediated immune cell remodeling and Cytokine/Exosome signaling networks in the bone metastatic microenvironment. This figure depicts how tumor cells reshape immune-cell function and build an immunosuppressive network via cytokines and extracellular vesicles (EVs) within the bone metastatic niche. (**Upper panel**)—tumor-mediated immune-cell remodeling: tumor-secreted PTHrP, TGF-β, IL-10, CCL2, CSF-1 and lactate trigger monocyte-to-M2-type tumor-associated macrophage (TAM) polarization, CD8^+^ T-cell exhaustion (PD-1, TIM-3, LAG-3, TIGIT upregulation), enrichment of regulatory T (Treg) and B (Breg) cells, and natural killer (NK)-cell dysfunction. NK-cell impairment arises from ADAM10/17-dependent MICA/B shedding and NKG2D downregulation. TAMs amplify immunosuppression by expressing VEGF, MMP-9 and PD-L1. (**Lower panel**)—cytokine and exosome signaling networks: Primary/metastatic tumors remotely remodel bone marrow using soluble mediators (IL-1β, IL-6, TGF-β, CCL2, CXCL12) to induce chronic inflammation and recruit immunosuppressive cells. Tumor-derived exosomes containing miRNAs and lncRNAs drive pre-metastatic niche (PMN) formation through extracellular matrix (ECM) remodeling and elevated vascular permeability. Together, these pathways construct a strongly immunosuppressive bone microenvironment enabling tumor colonization and progression.

**Figure 2:**
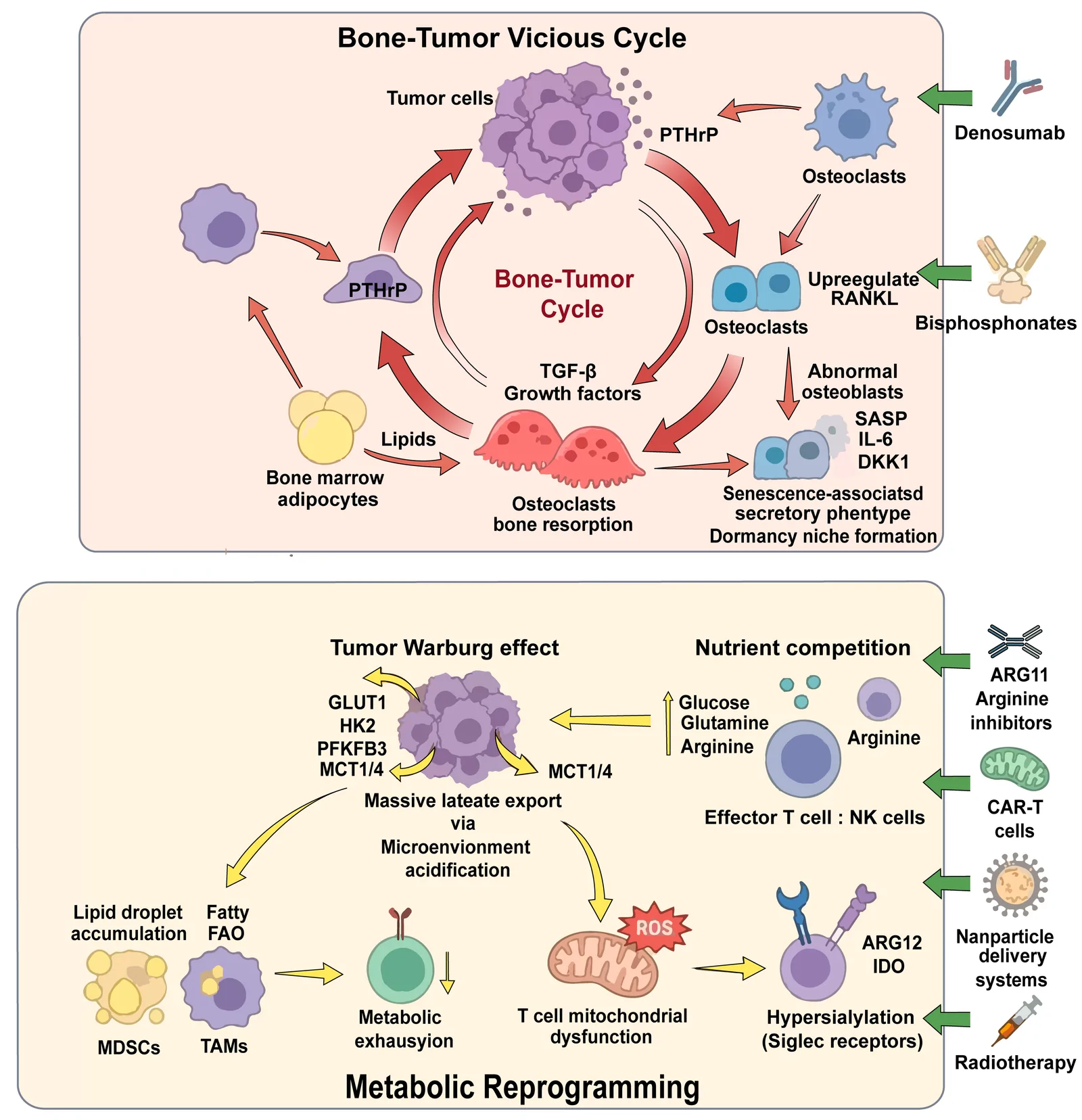
The bone-tumor vicious cycle and metabolic reprogramming in bone metastasis. This figure illustrates reciprocal crosstalk among tumor cells, bone-resident cells, and metabolic pathways driving bone metastasis progression and immune evasion. Figure created using Adobe Illustrator. (**Upper panel**)—bone-tumor vicious cycle: tumor-secreted PTHrP induces osteoblastic RANKL expression to activate osteoclast-mediated bone resorption. Bone-matrix-released TGF-β further promotes tumor proliferation, establishing the canonical “vicious cycle”. Denosumab (anti-RANKL antibody) and bisphosphonates suppress osteoclast activity to interrupt this cycle. Dysfunctional osteoblasts drive progression via senescence-associated secretory phenotype (SASP), IL-6/DKK1 secretion, and dormancy-niche formation for tumor immune escape. Bone-marrow adipocytes supply lipid substrates supporting tumor and immune-cell metabolism. (**Lower panel**)—metabolic reprogramming: tumor Warburg effect up-regulates GLUT1, HK2 and PFKFB3 to boost glucose uptake; lactate exported through MCT1/4 causes microenvironmental acidification. Tumor-stromal ARG1/2 and glutaminase (GLS) deplete arginine and glutamine, inducing nutrient competition that impairs effector T-cell and NK-cell function. Myeloid-derived suppressor cells (MDSCs) and tumor-associated macrophages (TAMs) accumulate lipid droplets and utilize fatty-acid oxidation (FAO), while T-cells develop mitochondrial dysfunction and exhaustion. Tumor-surface hypersialylation engages inhibitory Siglec receptors as a glycometabolic immune checkpoint. Green arrows denote candidate therapeutic approaches: arginase inhibitors, IDO inhibitors, nanoparticle delivery systems, CAR-T cells, and radiotherapy.

In the upper left panel, tumor-derived factors (PTHrP, TGF-β, IL-10, CCL2, CSF1, and lactate) lead to the polarization of monocytes into M2-type TAMs, exhaustion of CD8 T-cells (PD1, TIM-3, LAG-3, and TIGIT), enrichment of Tregs and Bregs, and impaired function of NK cells. The lower-left panel depicts the way in which primary and metastatic tumors remodel bone marrow remotely, through the secretion of cytokines such as IL-1β, IL-6, TGF-β, CCL2, and CXCL12, and through the secretion of exosomes carrying miRNA and lncRNA that form the pre-metastatic niche (PMN).

As shown in the upper right panel, the classic vicious circle is that a tumour-secreted PTHrP drives RANKL expression on osteoblasts, which activates osteoclasts and releases TGF-β to further stimulate tumour growth, and bone marrow adipocytes ((BAMs) provide a source of lipids while abnormal osteoblasts create dormancy niches through SASP. The bottom-right panel emphasizes metabolic reprogramming, where tumor cells take up glucose (Warburg effect) and produce and release lactate, leading to acidification and a deficiency of nutrients, thereby depleting effector T and NK cells, while MDSCs and TAMs utilize fatty acid oxidation and lipid accumulation.

### 3.1 Tumor-Cell-Mediated Remodeling of Immune Cell Function

Bone is the most common site of distant metastasis for malignant tumors, and its tumor microenvironment (TME) is highly heterogeneous and complex. Once tumor cells enter the bone marrow cavity, they not only activate osteoclasts by secreting factors such as parathyroid hormone-related protein (PTHrP), triggering a “vicious cycle” in which bone destruction and tumor growth mutually reinforce each other, but also actively induce functional remodeling in infiltrating immune cells through metabolic remodeling and cytokine networks, transforming the immune system from a “surveiller” into an “accomplice” to tumor growth, thereby contributing to the establishment of a deeply suppressed immune niche.

#### 3.1.1 Imbalance in Macrophage Polarization

During the progression of bone metastases, macrophages exhibit a high degree of phenotypic plasticity. Bone marrow stromal cells and tumor cells secrete colony-stimulating factor-1 (CSF-1) and the chemokine CCL2, which recruit large numbers of peripheral monocytes to migrate to the site of bone metastasis. Under normal physiological conditions, macrophages tend to polarize into the M1 phenotype, which exhibits antitumor activity; however, high levels of transforming growth factor-β (TGF-β), interleukin-10 (IL-10), and lactate produced by tumor cell glycolysis within bone metastases collectively have been shown to promote macrophage polarization toward the pro-tumor M2 phenotype (i.e., tumor-associated macrophages, TAMs) [3,4].

The aforementioned polarization imbalance has specific pathological significance in the bone microenvironment. On the one hand, M2-type TAMs induce abnormal vascular proliferation within the bone marrow by secreting vascular endothelial growth factor (VEGF), thereby continuously supplying nutrients for tumor growth; on the other hand, matrix metalloproteinase-9 (MMP-9) and collagenase secreted by TAMs can directly degrade the bone matrix, helping tumor cells carve out invasion pathways through mineralized bone tissue. Furthermore, TAMs can directly interact with T cells by highly expressing programmed death-ligand 1 (PD-L1), releasing inhibitory signals and thereby establishing the first line of immunological and physical defense at the site of bone metastasis [5,6].

#### 3.1.2 T-Cell Functional Exhaustion and Subpopulation Imbalance

T cells are the core effector cells of the body’s antitumor immune response; however, in bone metastases, their function is impaired, manifesting as a dual characteristic of “functional exhaustion” and “subpopulation imbalance.” Under the synergistic effects of chronic antigen stimulation and the hypoxic microenvironment of the bone marrow, CD8^+^ cytotoxic T cells enter a state of progressive exhaustion, characterized by the co-upregulation of multiple inhibitory receptors, including PD-1, TIM-3, LAG-3, and TIGIT [8]. When PD-L1, which is upregulated on the surface of tumor cells, binds to PD-1 on T cells, it leads to altered activity of key transcription factors (such as NFAT) in intracellular signaling pathways, causing CD8^+^ T cells to lose their ability to secrete interferon-γ (IFN-γ) and tumor necrosis factor-α (TNF-α), and significantly weakening their ability to lyse and kill tumor cells [7,9].

At the same time, there is a marked enrichment of immunosuppressive cell subsets within bone metastases. High concentrations of TGF-β released during osteoclast-mediated bone resorption serve as a key signal driving the differentiation and expansion of regulatory T cells (Tregs). By secreting IL-35 and TGF-β, Tregs not only directly suppress the activation and proliferation of effector T cells but also impair the functional maturation of DCs. Studies have shown that the ratio of Tregs to CD8^+^ T cells is severely inverted within bone metastases, and this subpopulation imbalance is one of the key reasons for the development of resistance to immunotherapy in patients with bone metastases [10,11].

#### 3.1.3 Abnormal Function of B Cells and NK Cells

Recent studies have revealed the underlying mechanisms by which B cells and NK cells contribute to immune remodeling in bone metastases. At the B-cell level, the bone marrow—the primary site of B-cell development—undergoes profound disruption of its homeostasis following tumor invasion. Emerging evidence suggests that tumor cells induce the conversion of bone marrow B cells into regulatory B cells (Bregs) through signaling mediated by EVs. Bregs highly express granzyme B and IL-10, which can directly induce apoptosis in effector T cells and, in synergy with TGF-β, further promote the expansion of Tregs, thereby amplifying the local immunosuppressive effect [12,13]. While these mechanisms have been most extensively characterized in other tumor types, emerging evidence suggests similar pathways are active in the bone metastatic niche, although further studies are needed to confirm their relative contributions.

At the NK cell level, NK cells—which serve as part of the innate immune defense—often exhibit significant functional “blinding” in bone metastases. Tumor cells cleave the ligands MICA/B on their surface—which activate the receptor—using metalloproteases (ADAM10/17), producing large amounts of soluble sMICA. The latter competitively binds to the NKG2D receptor on the surface of NK cells via a “decoy” mechanism, inducing the internalization and degradation of NKG2D, thereby blocking the activation signals of NK cells. Furthermore, the highly acidic metabolic environment within bone metastases significantly inhibits mitochondrial energy metabolism in NK cells, severely impeding the degranulation process of perforin and granzyme, ultimately causing NK cells to lose their critical role in the early elimination of circulating tumor cells (CTCs) and minimal residual disease (MRD) in the bone marrow [14,15].

Overall, tumor cells have created a multilayered, very sophisticated immune evasion program in the unique microenvironment of bone tissue by synergistic remodeling of macrophages, T cells, B cells, and NK cells. The challenge for future research is how to devise a method to specifically target this inhibitory microenvironment with small-molecule inhibitors or monoclonal antibodies. The simultaneous targeting of bone metabolism and immune checkpoint pathways can offer a way to disrupt the immune homeostasis of bone metastases, thus providing the theoretical basis for novel advances in the immunotherapy of bone metastases.

### 3.2 The Role of Immune Regulation in the “Vicious Cycle” between Bone and Tumor Cells

In the pathological process of bone metastasis, the “vicious cycle” of tumor cells and bone resident cells (mainly osteoclasts and osteoblasts) is the key mechanism in bone progression and destruction. The traditional view is that the factors secreted by the tumor cells promote the activation of osteoclasts, which leads to bone resorption, through the upregulation of the expression of receptor activator of nuclear factor κB ligand (RANKL) by osteoblasts. During the bone matrix degradation process, osteoclast-mediated resorption releases high concentrations of growth factors, including TGF-β, which in turn promote tumor cell proliferation and survival—a hallmark of the bone metastasis “vicious cycle” [16,17]. The latest advances on the bone immunology field show that this “vicious cycle” has a significant impact on the bone marrow immune microenvironment, where metabolic reprogramming of BMAs supplies lipid energy to tumor cells, contributing to tumor establishment, as well as immune metabolic homeostasis in the bone marrow [18]. Within the bone marrow, tumour cells, bone-resident cells and immune cells (T cells, macrophages, MDSCs, etc.) interact in an intense manner and in multiple directions, through the action of cytokine networks, to create strong local immune suppression, which enables tumour cells to successfully escape from the host immune system surveillance [4].

#### 3.2.1 Immunosuppressive Effects of Osteoclast Activation

Osteoclasts not only play a direct role in bone resorption, but are also important agents of the immunosuppressive environment in the bone metastasis microenvironment.

The functional role of homology and interaction between osteoclasts and immune cells.

Osteoclasts are derived from the mononuclear phagocytic system and share a common precursor with macrophages and DCs from the myeloid lineage. MDSCs can directly differentiate into mature osteoclasts, thus creating bone resorption and enhancing local immunosuppression—a twofold effect—in the microenvironment of bone metastases under specific conditions [19]. This bidirectional “immune-bone metabolism coupling mechanism causes immunosuppression and bone destruction in bone metastases to mutually reinforce each other, making them inseparable.

(1) A large number of immunosuppressive factors are released in the process of bone resorption

Bone matrix breakdown by osteoclasts leads to high release of TGF-β. Although TGF-β is a potent inhibitor of the cytotoxic activity of cytotoxic T lymphocytes (CTLs) and NK cells, it also stimulates the growth of Tregs, and thus effectively inhibits the adaptive antitumor response as a whole [20]. The effects together form a positive feedback loop of bone resorption and immunosuppression with a central role for TGF-β.

(2) Directly causing immune cell exhaustion

Osteoclast activation is accompanied by continued activation of the NF-κB and MAPK signaling pathways, which can further increase the local inflammatory response and contribute to rapid functional exhaustion of immune cells with production of IL-6 and IL-8 cytokines [17]. It is noteworthy to mention that the Tregs in the bone microenvironment can produce an inhibitory factor to exert immunoregulatory effects, but they are also a source of RANKL, which exerts a direct effect on osteoclastogenesis and forms a vicious positive feedback loop: “Tregs → osteoclasts → TGF-β → Tregs” [16].

#### 3.2.2 Immunoregulatory Effects of Abnormal Osteoblast Function

Osteoblasts play a fundamental role in maintaining bone homeostasis and the hematopoietic stem cell niche; however, in the context of tumor metastasis, their functional abnormalities can produce far-reaching immunoregulatory effects.

(1) Reshaping the myeloid suppression network

Tumor-derived soluble factors drive osteoblasts to secrete large amounts of IL-6, VEGF, and RANKL, which not only promote the recruitment and accumulation of MDSCs in the bone marrow but also upregulate their arginase 1 (ARG-1) expression, thereby exacerbating metabolic suppression of T cells through local arginine depletion [19]. Furthermore, factors secreted by osteoblasts, such as DKK1, have been shown to further enhance the immunosuppressive activity of MDSCs [20]. In addition to these soluble mediators, osteoblasts contribute to the local immunosuppressive milieu through mechanisms that are distinct from the TGF-β-driven effects described in Section 3.2.1, including the establishment of a dormancy niche (discussed below) and the senescence-associated secretory phenotype (SASP).

(2) Promotion of Tumor Dormancy and Immune Evasion

Dysfunctional osteoblasts can induce disseminated tumor cells to enter a cell cycle quiescent state by establishing a dormancy niche, thereby enabling them to evade immune recognition and killing by NK cells and CD8^+^ T cells, and providing a “sanctuary” for long-term latency of tumor cells [4].

(3) The Interfering Effects of the SASP

In a tumor-induced chronic inflammatory environment, osteoblasts can exhibit senescence-like characteristics and activate the SASP. The pro-inflammatory factors released by SASP (such as IL-1β and IL-6) not only suppress the maturation and antigen-presenting function of DCs but also promote the polarization of macrophages toward the M2 phenotype, thereby undermining the antitumor immune defense system on multiple levels.

(4) Weakening of Immune Function by Metabolic Reprogramming

During the process of bone metastasis, the abnormal proliferation of BMAs combines with microenvironmental acidification (primarily manifested as lactate accumulation) caused by impaired osteoblast function. Together, these factors synergistically limit the survival and functional maintenance of effector T cells, further reinforcing the immunosuppressive barrier at the metabolic level [18].

### 3.3 Signaling Networks Mediated by Cytokines and EVs

During the complex evolution of the bone metastasis microenvironment, intercellular communication relies on two parallel and interconnected signaling systems: (1) soluble cytokine and chemokine networks that mediate paracrine and endocrine signaling, and (2) EV-mediated communication that enables long-distance, targeted delivery of bioactive molecules across physical barriers [21]. Traditional thinking considered tumors to be local lesions, but emerging evidence indicates that tumors systematically pre-condition and remotely remodel the bone marrow—a “reserve of hematopoiesis and immunity”—by continuously releasing these signaling mediators even before bone colonization and during the early phases of bone metastasis [22]. In the cytokine network, chemokines and interleukins play a major role in maintaining the local inflammatory state and the systemic recruitment of immunosuppressive cells. In parallel, the lipid bilayer of EVs (particularly exosomes) enables them to stably transport and deliver nucleic acids (such as miRNAs and lncRNAs) and functional proteins to bone-resident cells and immune cells, creating an exquisite “Trojan horse” delivery effect [23,24]. The synergistic interplay between these two forms of communication—soluble chemical signaling and vesicular transport across physical barriers—substantially reprograms the original immune balance of the bone marrow, leading to its substantial reprogramming to a state of immune tolerance and tumor promotion. The following two subsections will address these two communication systems in detail: Section 3.3.1 focuses on the cytokine network, and Section 3.3.2 focuses on EV-mediated signaling.

#### 3.3.1 Immunosuppressive Effects of the Core Cytokine Network

The cytokine network serves as a central hub in the bone metastasis microenvironment that mediates chronic inflammation, drives the phenotypic transformation of immune cells, and facilitates immune evasion.

(1) Amplification of the Inflammatory Cascade via the IL-1 Signaling Pathway

In solid tumors with a high tendency for bone metastasis, such as breast cancer, the pro-inflammatory cytokine interleukin-1 (IL-1), particularly IL-1β, plays a central regulatory role in reshaping the inflammatory microenvironment. Tumor-derived IL-1β activates corresponding receptors on the surface of bone marrow stromal cells, osteoblasts, and vascular endothelial cells via autocrine and paracrine mechanisms, inducing a cascade-like release of secondary chemokines and inflammatory mediators [25]. This IL-1-driven chronic, persistent inflammation not only directly enhances tumor cells’ resistance to apoptosis and their invasive and migratory capabilities but also creates a microenvironment locally resembling a “chronic, non-healing wound,” providing the pathological basis for the subsequent establishment of an immunosuppressive network.

(2) Chemotactic Recruitment and Functional Cascade Activation of MDSCs

MDSCs are the core effector cell population in the immunosuppressive microenvironment of bone metastases. Tumor cells, by highly expressing CCL2 (MCP-1) and CXCL12 (SDF-1), recruit large numbers of monocyte-derived MDSCs (M-MDSCs) and granulocyte-derived MDSCs (PMN-MDSCs) from the bone marrow hematopoietic stem cell pool via the CCL2-CCR2 and CXCL12-CXCR4 receptor-ligand axes [26]. Upon arrival at the metastatic sites, these immature bone marrow-derived cells are rapidly activated by cytokines such as GM-CSF and IL-6 in the microenvironment, which trigger intracellular STAT3 and NF-κB signaling pathways. Deeply activated MDSCs subsequently upregulate the expression of arginase 1 (ARG-1) and inducible nitric oxide synthase (iNOS) and release large amounts of reactive oxygen species (ROS), leading to severe proliferative arrest and functional exhaustion of effector T cells by depleting local essential amino acids and inducing oxidative stress [20,26].

(3) Synergistic Immune Reprogramming by Multifunctional Cytokines

In addition to recruiting immunosuppressive cells, the synergistic action of TGF-β, IL-6, and IL-10 also exerts comprehensive functional suppression on existing antitumor immune cells. High concentrations of TGF-β released during bone matrix degradation downregulate the expression of MHC class II molecules on the surface of DCs, thereby blocking their antigen-presenting function; simultaneously, they inhibit the synthesis of perforin and granzyme in natural killer (NK) cells [20]. More critically, this combination of cytokines strongly induces naive CD4^+^ T cells to deviate from Th1 differentiation and instead polarize extensively into immunosuppressive Tregs and Th2 cells, thereby establishing a robust barrier of acquired immune tolerance within the bone microenvironment [27].

Notably, beyond their well-documented immunomodulatory roles, chemokine-centered signaling cascades triggered by genotoxic stress from chemotherapy and radiotherapy can promote tumor cell survival via immunity-independent mechanisms. Even in the absence of specific functional immune cells, bone-resident stromal fibroblasts are capable of releasing protective paracrine signals to drive stress-provoked epigenetic reprogramming, which sustains long-term tumor persistence. Tumor cells and immune cells can co-opt identical transcriptional regulators such as NF-κB to rewire their transcriptomic profiles upon therapeutic challenge. This adaptive stress response gives rise to acquired therapeutic resistance. Of translational importance, non-toxic pharmacological inhibition of the Mediator kinase module has been proven to efficiently abrogate this category of drug resistance. Collectively, besides immune-cell-dominated immunosuppression, this stroma-derived, immunity-independent stress-response axis also deserves consideration when designing combinatorial therapeutic regimens for bone metastatic malignancies.

#### 3.3.2 EV-Mediated Long-Distance Immune Regulation

Small extracellular vesicles (sEVs, often referred to as exosomes when endosomal origin is confirmed, approximately 30–150 nm in diameter), acting as biological carriers of complex molecular information, enable the primary tumor to precisely and remotely remodel the bone marrow microenvironment via the systemic circulation.

(1) Pre-establishment of the Pre-metastatic Niche (PMN)

The occurrence of overt bone metastasis is not accidental but is based on the prior, systematic remodeling of the bone marrow microenvironment. From an early stage, the primary tumor continuously releases tumor-derived extracellular vesicles (TEVs) into the peripheral blood that possess bone marrow homing properties; homing molecules on their surface, such as integrins, mediate the directed accumulation of these vesicles in the bone marrow. The angiogenic factors, MMPs, and integrin family proteins such as CD151 carried by these vesicles can rapidly initiate local extracellular matrix (ECM) remodeling and degradation, and upregulate local microvascular permeability [21,22]. This process is accompanied by the recruitment of a large number of inflammatory cells and the displacement of normal hematopoietic stem cells, thereby pre-establishing a highly protective pre-metastatic niche (PMN) for the homing and colonization of circulating tumor cells (CTCs) [21].

(2) Epigenetic Regulation of Osteoclast Differentiation and Immune Homeostasis by Non-coding RNAs

Non-coding RNAs (ncRNAs) are key cargo molecules in tumor-derived EVs that exert significant regulatory effects. Studies have confirmed that breast cancer-derived exosomes can efficiently deliver specific lncRNAs (such as lncRNA-MIR193BHG) to bone marrow monocytes and macrophages. Through a competitive endogenous RNA (ceRNA) mechanism, this lncRNA binds to miR-489-3p, thereby lifting its inhibition of the downstream DNA methyltransferase 3A (DNMT3A), and significantly promotes the differentiation and activation of osteoclast precursor cells into mature osteoclasts at the epigenetic level [28]. This EV-mediated abnormal osteoclast activation not only exacerbates osteolytic destruction but also further compromises the bone marrow immune microenvironment through the concomitant release of a large number of immunosuppressive factors [24].

(3) Vesicle-Mediated Remodeling of Macrophage Polarization

Oncogenic miRNAs (such as miR-21 and miR-155) carried by tumor-derived EVs can be internalized by bone marrow resident macrophages. By specifically inhibiting the translation of key negative regulatory genes such as PTEN or SOCS1, these miRNAs forcibly reprogram M1-type macrophages—which possess antigen-presenting and phagocytic functions—into M2-type TAMs that highly express CD206 and continuously secrete IL-10 and TGF-β, thereby significantly enhancing the immunosuppressive effects at the site of bone metastasis [23].

(4) Clinical Translation Prospects of Liquid Biopsy and EV-Targeted Interventions

Given the pivotal and critical role of EVs and their signaling networks in inducing bone marrow immune tolerance, they have become intervention targets of significant clinical value. On the one hand, quantitative analysis and content characterization of tumor-specific EVs (TEVs) in peripheral blood can serve as liquid biopsy biomarkers for early prediction of bone metastasis risk and dynamic monitoring of the immune microenvironment [21,22]; on the other hand, novel small-molecule inhibitors and neutralizing antibodies targeting the biogenesis, secretion, circulatory delivery, or uptake by target cells of EVs hold promise for blocking tumor-induced long-distance remodeling of the bone marrow microenvironment at its source, disrupting PMN formation, and opening new strategic pathways to enhance the therapeutic efficacy of ICIs in patients with advanced bone metastases [22,27].

### 3.4 The Effects of Metabolic Reprogramming on the Remodeling of the Immune Microenvironment

During the pathological progression of bone metastases, metabolic reprogramming has been established as a core driving mechanism by which tumor cells adapt to the extreme microenvironment of the bone marrow and induce profound immune tolerance. The bone marrow microenvironment is actually a very intricate environment made up of bone matrix cells, hematopoietic cells, and adipocytes. The bone marrow microenvironment is characterized by physiological hypoxia (low oxygen tension) and substantial lipid stores. Once tumour cells have colonised bone, their greatly increased anabolic requirements completely upset the local metabolic homeostasis. Tumor cells compete for scarce metabolic resources in the bone marrow (BM) by increasing the levels of enzymes and nutrient transporters that lead to predatory uptake of limited metabolic resources, thereby creating metabolic competition against infiltrating antitumor immune cells [29,30]. This systemic metabolic imbalance can cause a nutrient deficiency and metabolic stress for effector immune cells and is associated with the local accumulation of toxic metabolic byproducts (e.g., lactate, reactive oxygen species (ROS), kynurenine) in the microenvironment [31,32]. These metabolic changes are not just those of ordinary energy metabolism; they are the principal substrates that drive epigenetic changes and are intracellular signaling molecules that can drastically alter the transcription programmes and phenotypes of host immune cells to form a powerful barrier of immune suppression at the metabolic level.

#### 3.4.1 Metabolic Advantage of Tumor Cells and Immunosuppression

Through long-term evolution, tumor cells have acquired high metabolic plasticity. By remodeling the metabolic pathways of major macronutrients, they maintain their own malignant proliferation while systematically depriving surrounding immune cells of nutrients and subjecting them to toxic interference.

(1) Overexpression of Aerobic Glycolysis and the Glucose-Deprivation Effect

According to the classic Warburg effect, tumor cells remain highly dependent on the glycolytic pathway even under conditions of ample oxygen supply. In the bone metastasis microenvironment, tumor cells, through the synergistic activation of HIF-1α and c-Myc, significantly upregulate the expression of glucose transporters (such as GLUT1) and key rate-limiting enzymes (such as hexokinase HK2 and phosphofructokinase PFKFB3), enabling highly efficient competitive uptake of glucose from the microenvironment and leading to a sharp drop in local glucose concentration [29,32]. Since CTLs are also highly dependent on ATP derived from glycolysis during antigen activation and the execution of effector functions—to meet the energy demands of cell proliferation and the synthesis of effector molecules such as IFN-γ and perforin—tumor-induced extracellular glucose deprivation can directly block the activation of the mTORC1 signaling pathway within T cells, forcing them into cell cycle arrest and causing them to lose their effector function [29,30].

(2) Signal inhibition mediated by massive lactate efflux and microenvironmental acidification

Intense glycolytic activity in tumor cells inevitably leads to the production of large amounts of lactate. To avoid intracellular acidosis, tumor cells use monocarboxylate transporters 1 and 4 (MCT1/MCT4) to pump large amounts of lactate and protons out into the extracellular matrix, lowering the local microenvironment pH to 6.0–6.5. High concentrations of extracellular lactate, on the one hand, alter the transmembrane concentration gradient, hindering T cells and NK cells from excreting the lactate produced by their own metabolism, thereby triggering a feedback-mediated inhibition of glycolysis; on the other hand, as a bioactive signaling molecule, lactate directly inhibits the phosphorylation of p38 MAPK and JNK kinases within T cells, disrupting the nuclear translocation of the transcription factor NFAT, thereby fundamentally weakening TCR-mediated immune activation at the signaling level [32,33]. Microenvironmental acidification to pH levels of approximately 6.0–6.5 has been observed in a range of solid TMEs and is considered a general feature of tumor metabolic reprogramming, though direct pH measurements in the bone metastatic niche are limited.

(3) Competitive Depletion of Amino Acid Metabolism and Immunosuppressive Remodeling

In addition to glucose, tumor cells also exhibit significant metabolic dependence on specific amino acids such as glutamine and arginine. Bone-metastatic tumor cells highly express glutaminase (GLS), extensively consuming glutamine from the microenvironment to replenish the tricarboxylic acid (TCA) cycle and support macromolecular synthesis, thereby directly impairing the redox balance and proliferative capacity of T cells [31]. At the same time, tumor cells and the stromal cells they recruit deplete arginine and tryptophan in the microenvironment, respectively, through the overexpression of arginase 1 (ARG1) and indoleamine 2,3-dioxygenase (IDO). Kynurenine, a byproduct of tryptophan degradation catalyzed by IDO, binds to the aryl hydrocarbon receptor (AhR) in T cells, specifically inducing the differentiation and expansion of Tregs, thereby further enhancing immunosuppressive effects [29,31].

#### 3.4.2 Metabolic Reprogramming and Functional Abnormalities of Immune Cells

In response to the metabolic pressure from tumors, and to the constant presence of toxic metabolites, innate and adaptive cells in the bone marrow have to undergo metabolic adaptations that are cell-autonomous, but can often be associated with the loss of normal anti-tumor defense mechanisms.

(1) Mitochondrial Dysfunction and Metabolic Exhaustion in Effector T cells

During the prolonged sustained tumour antigen load and metabolic stress (e.g., hypoxia and nutrient deprivation), the CD8^+^ T cells infiltrating bone metastases gradually become terminally exhausted, a state known as “metabolic exhaustion” [34]. The basic molecular pathological characteristics of the disorder are characterized by a systemic disorder of mitochondrial biogenesis, such as reduced expression of PGC-1α, abnormal reduction of the mitochondrial membrane potential, and excessive accumulation of ROS [29,33]. Exhausted T cells, in order to survive under conditions of nutrient deprivation, need to switch their metabolism from glycolysis to fatty acid oxidation (FAO). The FAO pathway, however, can be used to generate ATP for basic survival purposes but not the transient high energy requirements that are needed for the production of effector molecules. One of the important metabolic pathways that contribute to T cell intrinsic and extrinsic resistance to immune checkpoint blockade (ICB) therapy is this metabolic imbalance [34].

(2) MDSC lipid metabolic bias and increased immunosuppression

The extensively proliferated MDSCs in the bone marrow microenvironment have a strong metabolic flexibility, unlike the metabolic defect that occurs in effector T cells, with a strong bias toward lipid metabolism. MDSCs living inside the bone marrow cavity utilize a large number of BMAs as lipid substrates, upregulating the expression of two important fatty acid transport receptors, CD36 and members of the fatty acid transport protein (FATP) family, to ingest excessive exogenous free fatty acids, which are then converted into lipid droplets (LDs) [35,36]. Excessive lipid droplet accumulation is not only a way to store energy, but one of the important metabolic markers associated with the functional maturation and immunosuppressive activity of MDSCs. Active fatty acid oxidation (FAO) is not only critical for MDSCs to generate plenty of ATP, but also directly contributes to MDSCs’ capacity to highly express ARG-1 and iNOS and to produce inhibitory mediators like peroxynitrite [35].

Distinct metabolic profiles of M-MDSCs versus PMN-MDSCs: Recent studies have revealed significant metabolic heterogeneity between the two major MDSC subsets in the bone marrow microenvironment. Monocytic MDSCs (M-MDSCs) exhibit a more pronounced reliance on oxidative phosphorylation (OXPHOS) and fatty acid oxidation (FAO), while granulocytic/polymorphonuclear MDSCs (PMN-MDSCs) demonstrate higher glycolytic activity. In the bone metastasis setting, M-MDSCs preferentially utilize bone marrow adipocyte-derived lipids via CD36-mediated uptake, sustaining their survival and immunosuppressive function through FAO-dependent ATP generation and NADPH production. In contrast, PMN-MDSCs rely more heavily on glycolysis and are less dependent on lipid metabolism. This metabolic divergence between subsets may reflect their distinct developmental origins and differential responses to the hypoxic, lipid-rich bone marrow microenvironment [35,36].

Lipid metabolism and MDSC longevity in the bone marrow: The lipid-rich bone marrow niche may prolong MDSC survival through several mechanisms. First, FAO-derived ATP supports the energetic demands of MDSCs in the nutrient-deprived, hypoxic bone marrow cavity, allowing them to persist longer than effector T cells that are metabolically disadvantaged under these conditions. Second, lipid droplet accumulation within MDSCs is associated with resistance to apoptosis and enhanced production of immunosuppressive mediators, including ARG-1 and iNOS. Third, bone marrow adipocytes secrete adipokines such as leptin and adiponectin, which further promote MDSC survival and immunosuppressive activity through signaling pathways including STAT3 and PI3K/AKT. This adipocyte-MDSC metabolic crosstalk represents an important but understudied mechanism contributing to the persistence of the immunosuppressive microenvironment in bone metastases [35,36].

(3) Polarisation and pro-tumoral reprogramming of the TAMs

Macrophages in the bone microenvironment are phenotypically polarized, and their polarisation is tightly controlled by underlying metabolic programmes. M1 macrophages are pro-inflammatory and cytotoxic, and depend heavily on glycolysis, PPP, and a disrupted TCA cycle for the production of ROS and nitric oxide, while M2 tumor-associated macrophages (TAMs) rely on an intact TCA cycle and oxidative phosphorylation (OXPHOS) to maintain their pro-tumor phenotype [37]. Lactate, cytokines, and a lipid-rich environment, secreted by the tumor cells, synergistically fuel M2 metabolic polarization by forcefully pushing osteoclast precursors and local macrophages in the direction of M2 metabolism in bone metastases [36]. This metabolic switch prevents macrophages from presenting and phagocytosing, and macrophages become great producers of VEGF and TGF-β, thus increasing the overall derangements of the bone marrow immune microenvironment [36,37]. Based on the above-described pathogenic mechanisms, multiple therapeutic modalities have been proposed to reverse bone-marrow immune tolerance, which are graphically summarized in Fig. 3.

**Figure 3:**
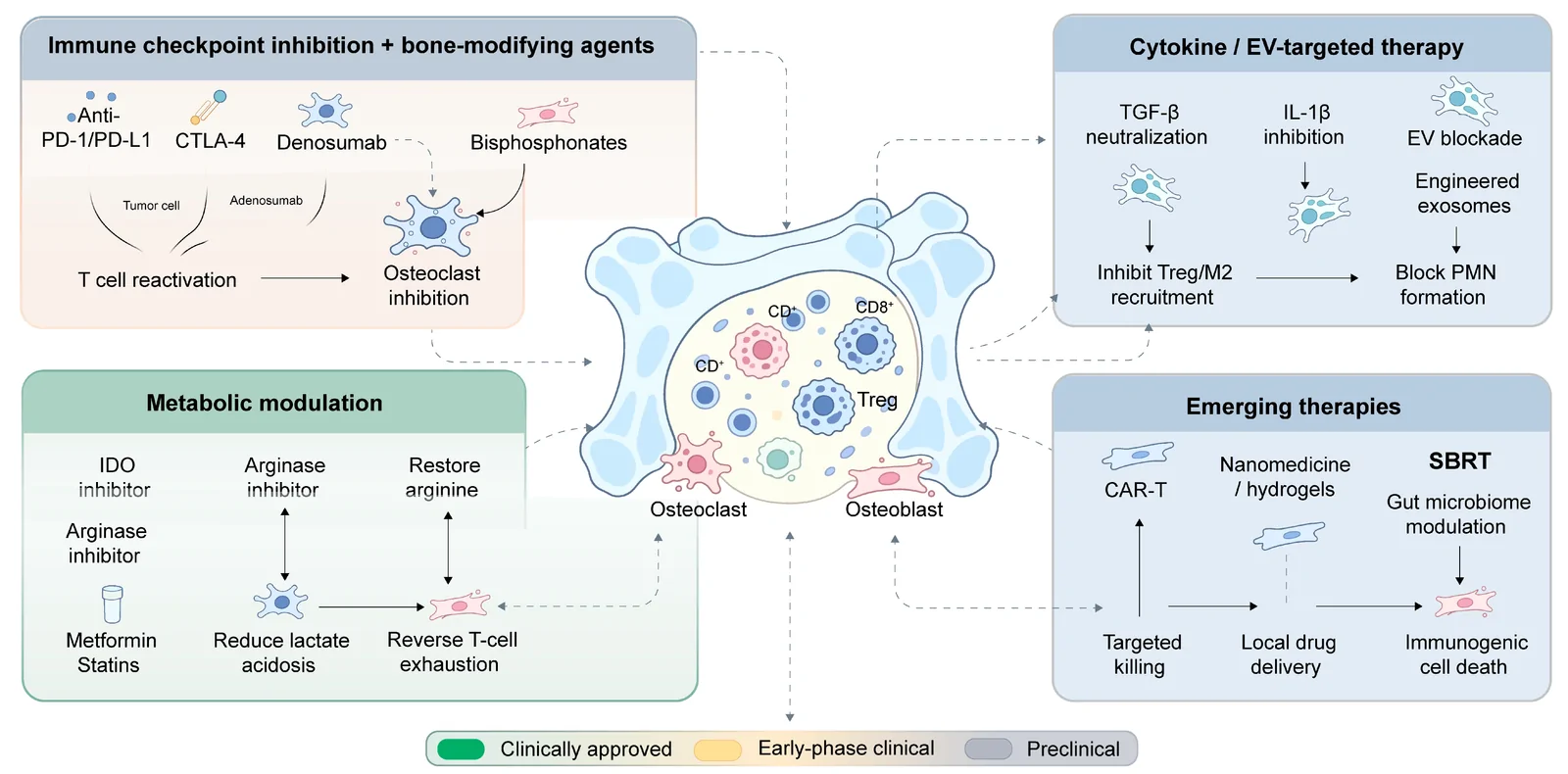
Therapeutic intervention strategies against immunosuppressive bone-metastatic niche.

Schematic overview of multi-modal therapeutic strategies targeting the immunosuppressive bone-metastatic microenvironment. This schematic summarizes intervention modalities including immune checkpoint inhibitors, bone-modifying agents, cytokine/extracellular-vesicle-targeted therapy, metabolic modulators, nanomedicine/hydrogel delivery systems, engineered CAR-T adoptive cell therapy, gut microbiome modulation, and local stereotactic body radiotherapy. Arrows indicate therapeutic targets corresponding to pathological nodes described in Fig. 1 and Fig. 2. Color-coded boxes denote preclinical, early-phase clinical, and clinically approved interventions, highlighting translational gaps between basic research and routine clinical application.

## 4 Targeted Regulatory Strategies for the Immunosuppressive Microenvironment of Bone Metastases

As illustrated in Fig. 3, targeted regulatory strategies for the immunosuppressive microenvironment of bone metastases have evolved. Targeted regulation of the immunosuppressive microenvironment of bone metastases has evolved from the traditional approach of simply inhibiting bone destruction to a new, comprehensive therapeutic paradigm aimed at restoring immune homeostasis and enhancing systemic antitumor responses. The bone marrow microenvironment serves as a “sanctuary” for tumor cells; the immunosuppressive factors (such as TGF-β and IL-10) enriched within it, combined with the large number of infiltrating suppressive immune cells (such as MDSCs and Tregs), collectively form a natural barrier to antitumor immunotherapy [4,20]. The core of current regulatory strategies lies in interrupting the vicious cycle among tumors, bone, and the immune system: by precisely targeting specific immune checkpoints to reverse T-cell exhaustion and synergistically applying bone-protective agents to improve the local bone immune microenvironment, thereby breaking the “vicious cycle” and improving the overall prognosis and quality of life for patients with bone metastases [8,38].

### 4.1 Treatment with ICIs

The clinical application of ICIs has completely transformed the treatment landscape for advanced cancer; however, the clinical response rate in bone metastases is generally lower than that in primary tumors and other visceral metastatic sites.

#### 4.1.1 The Adverse Impact of Bone Metastases on the Efficacy of ICIs

Multiple large-scale systematic reviews and meta-analyses have shown that bone metastases are an independent risk factor for poor prognosis in patients receiving ICIs. Taking non-small cell lung cancer (NSCLC) as an example, both overall survival (OS) and progression-free survival (PFS) are significantly shorter in patients with bone metastases than in those without [39]. This is most likely due to the special immunological composition of the bone marrow microenvironment: PD-1/PD-L1 or CTLA-4 inhibitors are less effective in generating a sufficient infiltration of bone marrow by effector T cells, and the high concentration of TGF-β that is produced during the resorption of the bone further dilutes the potentiating effect of ICIs systemically [39,40].

#### 4.1.2 Bone-Specific Immunosuppressive Mechanisms and Drug Resistance

Retrospective clinical studies indicate that even in melanoma—a tumor type relatively sensitive to ICIs—bone metastases often exhibit stronger characteristics of immune tolerance. This resistance stems from highly polarized M2 macrophages and large clusters of MDSCs in the bone marrow—both of which deplete key metabolic substrates and co-express various inhibitory checkpoint molecules such as LAG-3 and TIGIT, forming a local “immune cold barrier” [4,38]. Furthermore, the profound exhaustion of T cells in the bone microenvironment and the loss of their memory phenotype make it difficult for monotherapy with ICIs to effectively reactivate the antitumor immune response [4].

#### 4.1.3 Heterogeneous Responses and Therapeutic Challenges

There is significant heterogeneity in the response to ICIs among bone metastases originating from different primary tumors. The current challenges lie in identifying ICIs-sensitive subgroups among patients with bone metastases and developing strategies that synergistically enhance the delivery of ICIs deep into the bone marrow to overcome the physical barriers to immune cell infiltration posed by high intramedullary pressure and stromal fibrosis [8,20].

### 4.2 Combination of Bone-Modifying Agents and Immunotherapy

Bone-modifying agents have traditionally been used to prevent SREs, but the latest evidence suggests that they also possess significant immunomodulatory potential and exhibit strong synergistic effects with ICIs.

#### 4.2.1 Mechanism of Immune Synergism of Denosumab

Denosumab, as an anti-RANKL monoclonal antibody, indirectly affects RANK-positive cells (including DCs and T cells) by blocking RANKL-RANK engagement. Studies have confirmed that denosumab reduces the concentration of local immunosuppressive factors, promotes DC maturation and antigen-presentation function, and thereby reactivates the antitumor activity of T cells [41]. Furthermore, by improving the vascular integrity of damaged bone tissue, it promotes the homing and infiltration of circulating immune cells into metastatic sites [41].

#### 4.2.2 Bisphosphonates and Macrophage Reprogramming

Bisphosphonates such as zoledronic acid not only induce osteoclast apoptosis but also, by modulating the mevalonate pathway, have been reported to promote the polarization of tumor-associated macrophages (TAMs) from the pro-tumor M2 phenotype toward the anti-tumor M1 phenotype, as demonstrated in preclinical breast cancer models [42]. A small retrospective series in hepatocellular carcinoma patients with bone metastases suggested a potential synergistic benefit when bisphosphonates were combined with ICIs, but this observation requires confirmation in larger, prospective cohorts [43].

#### 4.2.3 Evidence for Clinical Benefits

A retrospective study by Mabrut et al. evaluated the impact of denosumab on the efficacy of ICIs in 268 patients with bone metastases from various solid tumors. In the overall cohort, no significant difference in overall survival (OS) or progression-free survival (PFS) was observed between patients receiving ICI alone and those receiving ICI plus denosumab. An exploratory subgroup analysis identified a potential survival benefit in a small cohort of 17 patients who received denosumab after the initiation of ICI therapy, a finding that is hypothesis-generating and requires prospective validation [44]. Therefore, current clinical evidence does not support a definitive survival advantage of combining denosumab with ICIs in the general population of patients with bone metastases.

#### 4.2.4 Clinical Optimization of Combination Strategies

The optimal timing of bone-modifying agent administration in combination with ICIs remains an unresolved clinical question. Although preclinical and translational studies suggest that improving bone microenvironment homeostasis may potentiate antitumor immunity, the retrospective sequencing signal reported by Mabrut et al. cannot be considered conclusive [44]. Prospective studies, including the ongoing DENIVOS phase II trial [45], are expected to provide more definitive evidence regarding both the efficacy and the optimal sequencing of this combination strategy. At present, bone-modifying agents (bisphosphonates and denosumab) remain established standard-of-care for the prevention of SREs, and their role as validated immunotherapeutic sensitizers has not been established.

In addition to efficacy considerations, several practical and safety-related questions remain unresolved. First, the optimal timing and duration of denosumab administration relative to ICI initiation have not been established. The exploratory sequencing signal reported by Mabrut et al.—suggesting potential benefit when denosumab was administered after ICI initiation—raises the hypothesis that concurrent administration may not be optimal, but this requires dedicated prospective investigation. Whether prolonged denosumab treatment (beyond SRE prevention duration) confers additional immunomodulatory benefit remains unknown.

Second, the safety profile of combination therapy must be carefully considered. Denosumab and bisphosphonates are associated with well-established adverse effects including hypocalcemia and osteonecrosis of the jaw (ONJ), particularly with prolonged use. When combined with ICIs, which themselves carry risks of immune-related adverse events (irAEs), the potential for additive or synergistic toxicity exists. Although retrospective series have not reported a significantly increased incidence of severe adverse events with combination therapy compared to ICIs alone, hypocalcemia requires particularly close monitoring in patients receiving denosumab, especially those with renal impairment or inadequate vitamin D supplementation.

Third, the current clinical evidence base is limited. As noted above, the favorable signal for denosumab-ICI combination derives primarily from retrospective analyses and exploratory subgroups. Prospective data remain scarce, with the ongoing DENIVOS phase II trial expected to provide more definitive evidence. Until prospective, adequately powered trials are completed, the combination of bone-modifying agents with ICIs should be considered investigational for the purpose of immunomodulation, and their use should be guided by established indications for SRE prevention rather than assumed immunotherapy sensitization.

### 4.3 Therapeutic Strategies Targeting Cytokine and EV Pathways

During the pathological process of bone metastases, both soluble cytokines and EVs, especially exosomes, build up paracrine and endocrine communication networks between tumor cells, bone marrow stromal cells, and immune cells. Such a network helps to establish and maintain local immune tolerance during tumor metastasis to the bone marrow, and it is also involved in the creation and upkeep of the pre-metastatic niche (PMN) before the formation of overt metastasis. Therefore, precisely targeting and intervening in these intercellular communication pathways has been regarded as one of the key strategies for reshaping the tumor immune microenvironment and overcoming resistance to existing immunotherapies [46]. In preclinical and clinical studies of various highly malignant tumors, inhibiting abnormal signal transduction or delivering therapeutic molecules via extracellular carriers has demonstrated significant potential for reversing immune suppression and restoring antitumor immune responses [23].

Exosomes (EVs with a diameter of approximately 30–150 nm) have become key carriers mediating long-distance, targeted cellular communication due to their ability to stably transport bioactive molecules such as nucleic acids, proteins, and lipids. Currently, therapeutic strategies targeting EVs have evolved into two complementary approaches: inhibiting endogenous pro-tumor exosomes and developing engineered exosome delivery systems.

(1) Blocking the Metastasis-Promoting and Immunosuppressive Functions of Tumor-Derived Exosomes

Tumor-derived exosomes can colonize the bone marrow via the systemic circulation in the early stages of bone metastasis, reprogramming resident bone cells and immune cells at the epigenetic and transcriptional levels through the specific miRNAs and lncRNAs they carry. Studies have shown that prostate cancer-derived EVs can profoundly remodel the pre-metastatic bone microenvironment via activation of the mTOR/RAB1A signaling pathway, accelerating the establishment and progression of bone metastases [47]; breast cancer-derived exosomes similarly play a central role in recruiting bone marrow PMNs and promoting osteolytic destruction [48]. Furthermore, vesicles secreted by cancer stem cells (CSCs) and rich in non-coding nucleic acids such as lnc-PDGFD or miR-155-5p can be taken up by fibroblasts in the microenvironment, downregulating the expression of tumor suppressor genes (such as ACOT12) in target cells, inducing fibroblast activation, and promoting the epithelial-mesenchymal transition (EMT) and stromal remodeling of tumor cells. Thus, molecular intervention with inhibitors that inhibit exosomes biogenesis, secretion or uptake by target cells is a crucial point for the disruption of pre-metastatic communication and preserve bone marrow immune homeostasis.

(2) Engineered Exosome-Targeted Delivery Systems

The natural lipid bilayer of exosomes is highly biocompatible, has a low immunogenicity, and superior tissue penetration, which makes them an ideal platform for a new generation of nanodrug carriers for bone-related diseases. Chemotherapeutic drugs or immunomodulatory nucleic acid molecules can be genetically engineered or chemically conjugated and targeted to the exosome surface with bone targeting peptides, resulting in high levels of locally targeted drugs and dramatically decreased levels of off-target toxicity systemically [49].

(3) Spatiotemporal Controlled Release Strategies Combining Biomaterials

The physical incorporation or chemical crosslinking of exosomes on biocompatible materials like three-dimensional (3D) hydrogels has become a research focus in bone tissue engineering and postoperative local tumor therapy. Such exosome-integrated hydrogel systems enable the spatiotemporally controlled release of active payloads at sites of bone defects or tumor resection. Not only do they demonstrate excellent potential for inducing bone tissue reconstruction and angiogenesis, but they also maintain the long-term homeostasis of an immune microenvironment that suppresses tumor recurrence by continuously regulating the release of local inflammatory mediators [50].

### 4.4 Combination of Metabolic Modulators and Immunotherapy

In the microenvironment of bone metastases and widespread advanced solid tumors, tumor-cell-mediated abnormal metabolic reprogramming not only meets the energy demands of malignant proliferation and invasion but also constructs a profound “metabolic immune barrier” in specific niches—such as the bone marrow cavity—by reshaping local nutrient concentration gradients and accumulating inhibitory metabolic byproducts. Under this extreme metabolic stress, monotherapy with ICIs often encounters primary or acquired resistance due to severe metabolic exhaustion and functional decompensation of effector T cells. Therefore, the rational combination of modulators targeting specific metabolic pathways with immunotherapy—to lift immune suppression from the dual dimensions of energy substrate supply and metabolic signal transduction—has become one of the most promising translational medical strategies for overcoming immune tolerance in advanced tumors.

#### 4.4.1 Targeted Intervention in the Amino Acid Metabolic Network and Immune Remodeling

One of the central metabolic pathways implicated in immune evasion in tumors is the deprivation of essential amino acids in the local microenvironment, such as the excessive consumption of L-arginine and tryptophan. In bone metastases, both the tumor cells and the MDSCs recruited by them abnormally overexpress ARG1, which results in the rapid consumption of extracellular arginine. This results in direct downregulation of the expression of the CD3ζ chain in the TCR complex expressed on the surface of infiltrating T cells and causes effector T cells to arrest in the G0/G1 phase and drastically reduces the secretion of antitumor cytokines like IFN-γ [51]. The oral arginase 1/2 inhibitor OATD-02 has shown the ability to restore physiological arginine levels and reverse T-cell metabolic exhaustion in preclinical models, and has now entered first-in-human Phase I clinical studies (NCT05759923). However, another arginase inhibitor, INCB001158, demonstrated pharmacodynamic activity but limited antitumor activity in its first-in-human study, highlighting the need for careful clinical development and patient selection [52]. Systemic inhibition of IDO has been extensively explored as a strategy to break tryptophan-mediated metabolic tolerance. However, despite promising preclinical data, the Phase III ECHO-301/KEYNOTE-252 trial evaluating epacadostat (an IDO1 inhibitor) in combination with pembrolizumab failed to demonstrate improved PFS or OS in patients with melanoma. This negative result underscores the complexity of the IDO pathway and the challenges of translating metabolic inhibitors into clinical immunotherapy combinations. The development of IDO inhibitors as combination partners for ICIs remains uncertain, and subsequent trials have been limited [53].

#### 4.4.2 Targeted Interventions against Tumor-Surface Sialylation Modifications

Metabolic reprogramming in tumor cells is not only reflected in the excessive activation of the glycolytic pathway but also profoundly alters the network of glycosylation modifications on the cell surface. Hypersialylation is a hallmark of abnormal tumor glycometabolism; sialic acid glycan structures densely covering the tumor surface can specifically bind to inhibitory Siglec receptors (such as Siglec-7/-9) on the surface of NK cells and effector T cells. By recruiting SHP-1/2, they transmit strong negative regulatory signals to immune cells, constituting a non-classical “glycometabolic immune checkpoint” [54]. By specifically removing sialic acid residues from the tumor surface using small-molecule inhibitors targeting sialyltransferases or engineered sialidases, it is possible not only to directly lift the contact-mediated inhibition of immune cells by tumor cells but also to promote the deep infiltration of effector lymphocytes by reshaping the chemokine secretion profile of the microenvironment, thereby providing a new dimension for enhancing the efficacy of protein-targeted ICIs [54].

#### 4.4.3 Translational Application of the “New Uses for Old Drugs” Strategy

Given the lengthy development timelines for novel metabolic inhibitors, the repurposing of approved drugs with known safety profiles has gained attention. Statins and biguanides have shown potential to modulate the TME in preclinical and some observational studies. For example, statins may influence lipid metabolism in MDSCs and TAMs, while metformin can activate AMPK and potentially reduce local hypoxia and acidification. However, the translation to clinical benefit remains unproven: the Phase III MA.32 trial did not demonstrate improved invasive disease-free survival with metformin in early breast cancer [55]. Therefore, while metabolic repurposing represents an attractive concept, its role as an “immunosensitizing” strategy in bone metastases remains largely investigational and requires prospective, bone-specific trials. By systemically improving local metabolic homeostasis in the bone marrow and eliminating metabolic barriers that hinder T-cell function, these drugs may potentially generate synergistic effects with PD-1/PD-L1 inhibitors, thereby opening a new, cost-effective pathway for comprehensive immunotherapy in patients with advanced bone metastases.

### 4.5 Other Novel Regulatory Strategies

With the convergence of cutting-edge technologies such as spatial omics, single-cell sequencing, and materials engineering, intervention strategies targeting the immunosuppressive microenvironment of bone metastases are moving beyond the scope of traditional pharmacology. In addition to classic immune checkpoint blockade and metabolic interventions, targeted delivery systems based on novel biomaterials, genetically modified adoptive cell therapies, systemic regulation of the host microbiome, and synergistic local radiation therapy collectively form multidimensional microenvironment remodeling strategies, offering entirely new therapeutic opportunities for addressing systemic immune tolerance in advanced, refractory bone metastases.

#### 4.5.1 Targeted Delivery Systems Mediated by Nanomedicine and Biomaterials

Bone metastatic lesions are typically accompanied by highly mineralized extracellular matrix (ECM) deposits and abnormal stromal fibrosis; their extreme physical hardness severely impedes the effective penetration of macromolecular immunotherapeutic drugs during systemic administration. To overcome this physiological barrier, localized targeted delivery systems based on nanotechnology and injectable hydrogels have become a research focus. Loading chemotherapeutic agents or immunomodulators onto bone-seeking nanocarriers not only significantly prolongs the *in vivo* half-life of the drugs but also enables local concentration enrichment at the lesion site through high affinity for the bone matrix [56]. Composite nanosystems based on functionalized nucleic acids (such as siRNA and aptamers) can precisely silence specific immunosuppressive genes within target cells post-transcriptionally, demonstrating profound gene regulatory capabilities in various highly malignant tumors [22]. Moreover, microsphere-composite hydrogels can be physically delivered as *in situ* delivery platforms and, through the release of specific chemokines, can attract mesenchymal stem cells and remodel the local macrophage population, thus facilitating the formation of healthy bone immune tissue while killing the tumor cells [57].

#### 4.5.2 Breakthroughs and Optimization of Engineered Adoptive Cell Therapy (CAR-T)

Although chimeric antigen receptor T-cell (CAR-T) therapy has achieved landmark success in hematologic malignancies, its efficacy in solid tumors—including bone metastases—remains constrained by the highly immunosuppressive microenvironment. The predominance of M2-polarized macrophages and the T-cell exhaustion network within the bone TME severely limit the survival, infiltration, and functional persistence of adoptively transferred cells [58,59]. In the context of bone metastases, the physical barrier posed by the mineralized bone matrix and the elevated interstitial pressure within the bone marrow cavity further impede CAR-T cell trafficking to tumor sites.

Preclinical studies in osteosarcoma models have provided proof-of-concept evidence for CAR-T therapy in bone-residing tumors. For example, Talbot et al. demonstrated that B7-H3-targeted CAR-T cells exhibited clear dose-dependent antitumor effects and precise killing capacity in orthotopic osteosarcoma models that recapitulate *in situ* bone colonization and spontaneous metastasis to the lungs [59]. However, several critical caveats must be emphasized. First, osteosarcoma is a primary bone sarcoma with distinct biological characteristics—including tumor cell origin, genetic drivers, antigen expression profiles, and microenvironment composition—that differ substantially from carcinoma bone metastases (e.g., those arising from breast, prostate, or lung cancer). Second, the cited study focused on pulmonary metastasis from osteosarcoma rather than bone metastasis from epithelial tumors, limiting the direct extrapolation of these findings to the clinical bone metastasis setting. Third, bone metastases from carcinomas exhibit greater tumor heterogeneity and a more complex immunosuppressive network, which may render CAR-T cells less effective than observed in sarcoma models.

To overcome these challenges and translate CAR-T therapy to carcinoma bone metastases, several strategies are under active investigation. Fourth-generation CAR-T cells engineered to co-express cytokines such as IL-15 are being developed to enhance persistence within the nutrient-deprived, hypoxic bone marrow microenvironment [58]. Combinatorial approaches with bone-selective matrix-modulating agents (e.g., bisphosphonates or collagenases) are being explored to disrupt the extracellular matrix barrier and facilitate CAR-T cell infiltration into deep osteolytic lesions [60]. In addition, dual-targeting CAR designs targeting multiple tumor-associated antigens may help address antigen escape, a common mechanism of resistance in heterogeneous solid tumors. Nevertheless, these approaches remain predominantly at the preclinical stage, and their translation to patients with carcinoma bone metastases faces substantial hurdles, including the identification of bone metastasis-specific surface antigens, management of on-target/off-tumor toxicity, and overcoming the profound local immune suppression unique to the bone marrow niche.

#### 4.5.3 Systemic and Local Immune Regulation by the Host Microbiome

The gut microbiota exerts a profound influence on distant metastasis of solid tumors via the “gut-bone axis” and systemic immune circulation. Metagenomic analyses reveal that specific microbial communities residing in tissues such as the gut and breast not only directly regulate the host’s baseline systemic inflammation through metabolites such as short-chain fatty acids but are also deeply involved in the initiation of bone metastasis [61]. Changes in the abundance of specific microbial communities can influence the lineage differentiation of hematopoietic stem cells in the bone marrow and even induce pre-osteoclast activation, thereby creating a pre-metastatic niche conducive to the colonization of circulating tumor cells. Furthermore, the intratumoral microbiome has been shown to modulate intracellular signaling cascades in host cells, affecting tumor sensitivity to anticancer drugs [62]. Therefore, reshaping the host microbial ecosystem through dietary interventions, targeted antibiotic selection, or fecal microbiota transplantation (FMT) is being developed as a novel adjuvant therapy to reverse systemic and local immune suppression and block the occurrence of bone metastases [61,62].

#### 4.5.4 Microenvironment Reprogramming and Immune Synergistic Effects Mediated by Local Radiation Therapy

For bone metastases characterized by high levels of immune tolerance, systemic immune checkpoint blockade alone often fails to produce sufficient antitumor effects. The introduction of local physical interventions, such as stereotactic body radiation therapy (SBRT), can induce immunogenic cell death (ICD) in tumor cells within the target area, accompanied by the massive exposure of tumor-associated antigens and the release of danger-associated molecular patterns (DAMPs), thereby effectively promoting the maturation and cross-presentation of intramedullary DCs. Combining radiation therapy-induced local immune activation with systemic anti-PD-1/CTLA-4 therapy not only transforms “immunologically cold tumors” within the bone marrow into “immunologically hot tumors,” but also holds promise for triggering an abscopal effect, leading to a sustained immune clearance response against microscopic metastases throughout the body [4].

### 4.6 Safety Considerations in Combination Strategies

The clinical translation of combination regimens targeting the bone metastatic microenvironment must also address potential safety concerns. ICIs are associated with immune-related adverse events (irAEs) affecting multiple organ systems. Combining ICIs with bone-modifying agents may increase the risk of hypocalcemia, particularly with denosumab, and osteonecrosis of the jaw (ONJ) remains a recognized complication of bisphosphonate and denosumab therapy. The addition of metabolic modulators or cytokine-targeting agents may further compound toxicity profiles. Therefore, careful patient selection, monitoring, and prospective safety assessments are essential in the design of future combination trials. The risk-benefit ratio of each combination strategy must be rigorously evaluated, particularly in patients with advanced bone metastases who may already have compromised performance status. A summary of all therapeutic strategies discussed above, categorized by evidence level, is provided in Table 1.

**Table 1:** Summary of therapeutic strategies targeting the immunosuppressive bone metastatic microenvironment by evidence level.

Therapeutic Strategy	Agent/Class	Target Pathological Mechanism	Evidence Level	Key Studies/Trials	Sample Size & Key Supporting Data	Limitations	References
Bone-modifying agents (SRE prevention)	Denosumab, Bisphosphonates	Inhibit osteoclast-mediated bone resorption; indirect immunomodulation via lowering TGF-β release	Approved	Phase III registration trials	>7000 patients across pivotal phase-III trials; significantly reduced skeletal-related events risk vs. control; no formal immunological endpoint	Not approved as immunotherapy sensitizer	[41]
Bone-modifying agents + ICIs	Denosumab + anti-PD-1/PD-L1	Block RANK-RANKL axis; promote DC maturation; combine with checkpoint blockade to reverse T-cell exhaustion	Retrospective, hypothesis-generating	Mabrut et al. 2024 (n = 268, exploratory subgroup n = 17)	Total cohort n = 268 solid-tumor bone-metastasis patients; overall cohort OS/PFS no statistical difference; exploratory subgroup n = 17 (denosumab after ICI initiation) suggested potential survival signal, no statistical validation	No overall survival benefit in whole cohort	[44]
Bone-modifying agents + ICIs	Denosumab + anti-PD-1/PD-L1	Block RANK-RANKL axis; promote DC maturation; combine with checkpoint blockade to reverse T-cell exhaustion	Phase II ongoing	DENIVOS (GFPC 06-2017)	Ongoing phase II trial in NSCLC patients with bone metastases; no mature clinical efficacy endpoints available	Only abstract-level interim information; final study results pending	[45]
IDO inhibitor + ICI	Epacadostat + pembrolizumab	Blocks tryptophan catabolism; reduces Treg differentiation driven by kynurenine	Phase III negative	ECHO-301/KEYNOTE-252	n = 706 unresectable melanoma patients; median PFS 4.7 months (combination) vs. 4.9 months (placebo + pembrolizumab); no OS improvement	No improvement in PFS or OS; trial conducted in melanoma patients, no dedicated bone-metastasis cohort	[63]
Arginase inhibitor + ICI	OATD-02	Restore extracellular arginine concentration; rescue TCR-complex function and reverse effector T-cell metabolic exhaustion	Phase I ongoing (NCT05759923)	Preclinical + Phase I	Pre-clinical *in-vivo* model: restored intratumoral arginine, enhanced CD8^+^T effector function; phase-I ongoing, no mature efficacy readout available	Early stage, limited efficacy data	[64]
Arginase inhibitor + ICI	INCB001158	Arginase-1/2 inhibition, relieve arginine-depletion-mediated T-cell suppression	Phase I completed, limited activity	First-in-human study	n = 103 advanced solid-tumor patients; target pharmacodynamic change observed, but objective response rate low (ORR < 5%)	Pharmacodynamic effect but limited antitumor activity	[52]
Drug repurposing (metabolic)	Metformin, Statins	Modulate lipid/glycolytic metabolism; reprogram MDSCs/TAM functional status	Preclinical/Observational	MA.32 trial (negative), observational studies	MA.32 n = 3649 early breast-cancer patients; metformin failed to improve invasive-disease-free survival; observational cohorts show correlative signal but no causal proof	Not validated in bone metastasis; prospective trials needed. Trial enrolled early-stage breast cancer without bone-metastasis patients	[65]
CAR-T therapy	B7-H3 CAR-T	Specific antigen-driven cytotoxic killing of tumor cells	Preclinical (osteosarcoma model)	Talbot et al. 2021	Orthotopic osteosarcoma mouse model: dose-dependent anti-tumor and anti-pulmonary-metastasis effect observed in mice	Model is osteosarcoma, not carcinoma bone metastasis	[59]
Local radiotherapy + ICI	SBRT + ICIs	Induce immunogenic cell death ICD; promote DC cross-presentation; convert immune-cold bone lesion into immune-hot phenotype	Preclinical/Early phase	Various retrospective series	Retrospective cohorts: improved local control rate; durable abscopal effect only observed in small subset of patients	Requires prospective validation	[4]

**Note:** Abbreviations: SRE, skeletal-related event; ICI, immune checkpoint inhibitor; PD-1, programmed cell death protein 1; PD-L1, programmed death-ligand 1; IDO, indoleamine 2,3-dioxygenase; PFS, progression-free survival; OS, overall survival; SBRT, stereotactic body radiation therapy.

## 5 Challenges and Outlook

Although targeted strategies for modulating the immunosuppressive microenvironment of bone metastases have demonstrated remarkable therapeutic potential in preclinical studies and some early-phase clinical trials, numerous scientific challenges and clinical hurdles still need to be overcome to achieve a comprehensive translation from basic mechanisms to routine clinical practice.

### 5.1 Spatiotemporal Heterogeneity of the Bone Immune Microenvironment: The Primary Obstacle to Precision Therapy

Bone metastasis is not a static pathological endpoint, but rather a highly dynamic evolutionary process. At different stages of tumor colonization (micrometastatic stage, dormant stage, and overt metastatic stage), there are significant differences in the cellular composition, metabolite concentration gradients, and the relative importance of immune-suppressive signaling networks within the bone marrow microenvironment. Furthermore, the patterns of immune infiltration exhibit extreme heterogeneity not only among different primary tumors (such as osteogenic metastases from prostate cancer and osteolytic metastases from breast cancer) but also among metastatic lesions in different bone sites within the same patient. Existing targeted strategies are often based on single targets or static mechanistic models, making it difficult to address this complex variability—which is both systemic and dynamic—and this represents the primary obstacle to achieving precise interventions.

### 5.2 Lack of Bone-Specific Predictive Biomarkers for Immunotherapy

The immunotherapy biomarkers currently in widespread clinical use—including PD-L1 expression rates, tumor mutation burden (TMB), and microsatellite instability-high (MSI-H)—are primarily derived from data on primary tumors or soft tissue metastases. However, decalcification of bone tissue may compromise the integrity of tissue antigens; coupled with the highly immunosuppressive nature of the bone marrow, the sensitivity and specificity of these markers in predicting the efficacy of ICIs in patients with bone metastases are significantly reduced. Therefore, there is an urgent need to develop novel, composite biomarker systems—based on peripheral blood biopsies (such as circulating tumor DNA, tumor-derived exosomes, and specific metabolite abundance assays) or non-invasive molecular imaging techniques—that can reflect bone marrow immune homeostasis in real time and predict responses to combination therapy.

### 5.3 Systemic Toxicity and Targeted Delivery Bottlenecks in Combination Therapy Strategies

To overcome deep immune tolerance, clinicians often employ multi-target, multi-pathway combination regimens (such as dual immune checkpoint blockade, or ICIs combined with metabolic inhibitors or anti-angiogenic agents). However, this nonspecific systemic immune overactivation significantly increases the risk of severe immune-related adverse events (irAEs). At the same time, the physical barrier formed by the highly mineralized extracellular matrix of bone tissue, along with abnormally elevated interstitial pressure within the bone marrow cavity, severely limits the effective penetration of large-molecule antibody drugs and adoptive immune cells into the deep regions of the microenvironment, constituting an unavoidable delivery bottleneck for current combination therapy strategies.

## 6 Future Directions

Despite the significant advances in understanding the immunosuppressive bone metastatic microenvironment, several challenges remain. First, the spatiotemporal heterogeneity of the bone immune microenvironment across different tumor types, disease stages, and anatomical sites demands high-resolution characterization. Second, the lack of bone-specific predictive biomarkers limits patient selection and response monitoring. Third, systemic toxicity and targeted delivery bottlenecks must be addressed to translate combination strategies into clinical practice. To overcome these challenges, several future directions are promising.

### 6.1 Integration of Single-Cell and Spatial Multi-Omics Technologies

Leveraging platforms such as single-cell RNA sequencing (scRNA-seq), spatial transcriptomics, and mass cytometry will enable high-resolution mapping of the spatial distribution and dynamic communication networks among tumor cells, bone-resident cells, and immune subsets within bone metastases. These cutting-edge technologies offer the unique advantage of preserving the spatial context of cell–cell interactions, allowing researchers to identify spatially restricted immune-suppressive niches that may drive local tumor progression and therapy resistance [2]. Furthermore, integrating multi-omics datasets with computational network modeling will facilitate the precise identification of key cell subsets and vulnerable molecular nodes driving local immune tolerance, thereby laying a systematic theoretical foundation for the discovery of novel intervention targets [20,38]. Longitudinal single-cell profiling of patient samples before, during, and after treatment could also reveal dynamic changes in the immune microenvironment that correlate with clinical outcomes, enabling the development of dynamic biomarkers for treatment monitoring and adaptive therapeutic strategies.

### 6.2 Translational Development of Bioinspired Nanodelivery Systems and Tissue Engineering

Future research should focus on developing smart nanocarriers and injectable hydrogel systems with bone-seeking properties and microenvironment responsiveness (e.g., low pH response, high enzyme activity response, or ROS-responsive release). While significantly reducing systemic off-target toxicity, these systems enable the precise delivery and spatiotemporally controlled release of immunomodulators, metabolic inhibitors, and engineered immune cells to osteolytic or osteogenic lesions, thereby overcoming the bottleneck of drug delivery to the bone marrow cavity at the physical level [56]. Bioinspired strategies—including cell membrane-coated nanoparticles, biomimetic mineralization, and surface modification with bone-homing ligands such as bisphosphonates and aspartate-rich peptides—offer promising avenues for enhancing targeting efficiency and improving therapeutic indices. Moreover, the integration of imaging-guided delivery systems could enable real-time monitoring of drug distribution and accumulation at metastatic sites, facilitating personalized dose adjustment and optimizing treatment outcomes.

### 6.3 Development of Highly Bionic, Humanized In Vitro Evaluation Models

The establishment of “bone marrow-on-a-chip” or organoid models using microfluidic chip technology and 3D bioprinting techniques, which integrate human bone matrix components, hematopoietic stem cells, immune cells, and tumor cells, represents a transformative approach for studying bone metastasis. These platforms can better mimic the immune-metabolic crosstalk and cellular interactions that occur in human bone metastasis, including the dynamic interplay between tumor cells and the bone microenvironment under physiological flow and mechanical cues [57]. By incorporating patient-derived tumor cells and autologous immune cells, these models can recapitulate inter-patient heterogeneity and enable the screening of patient-specific therapeutic responses. Such systems are partially replacing the use of traditional animal experiments and improving high-throughput drug screening and iterative optimization of combination therapies, and could significantly reduce the time from mechanism discovery to clinical validation. Furthermore, the combination of organoid models with advanced imaging and multi-omics readouts will provide unprecedented resolution for dissecting the molecular mechanisms underlying therapy resistance and immune evasion in bone metastases.

### 6.4 Biomarker-Driven Adaptive Clinical Trial Design

In future clinical research, the one-size-fits-all treatment paradigm should be discarded, and umbrella/basket trials should be designed according to patient-specific metabolic subtypes/bone immune profiles. These trials can be done with dynamic biomarker screening and grouping, leading to truly individualized and precise immunomodulatory approaches against bone metastases, which in turn leads to an efficient translation of relevant treatment regimens to routine clinical practice.

## 7 Conclusion

In conclusion, the evolution of bone metastases cannot be simply considered as an invasion of tumour cells and an imbalance in bone remodelling but as a systemic immunopathological event in which tumour cells interact very closely with bone cells (osteoblasts and osteoclasts), with populations of immune cells and with metabolic-cytokine networks. Tumor cells have been able to create a highly suppressive environment in the bone marrow, a natural environment of hematopoiesis and immunity, via paracrine signaling, metabolic predation and epigenetic modulation by exosomes.

An extensive understanding of the underlying mechanisms of this microenvironment—particularly the imbalance of macrophage polarization, metabolic exhaustion of T cells, and immunosuppressive mechanisms of the “tumor-bone” vicious cycle—has created a paradigm shift in the conceptualization of bone metastasis treatment. While traditional approaches that focus on symptom control through bone-resorption inhibitors are gradually being supplemented by more comprehensive targeted intervention strategies that aim to reverse immune suppression and reshape the local antitumor immune response, it is important to recognize that most of these novel combination strategies remain at the preclinical or early clinical stage.

The rational design of multidimensional combinations—targeting immune checkpoint pathways, combined with bone-protective agents, precision metabolic modulators, cytokine-neutralizing antibodies, nano-targeted delivery technologies, and adoptive cell therapy—holds theoretical promise to challenge the immune evasion strategies of bone metastases. However, the clinical evidence supporting these approaches is limited, and no combination strategy has yet been established as a standard of care for bone metastases.

While major hurdles exist in deep-level heterogeneity analysis, the identification of specific biomarkers, and targeted drug delivery, the underlying regulatory mechanisms of the bone metastasis immune microenvironment are slowly becoming clearer with the ongoing progress of spatial multi-omics technologies and tissue engineering. Nevertheless, the bone metastatic niche appears to be immunologically and metabolically distinct, and the clinical value of targeting this niche alongside immunotherapy remains unproven. Future research must prioritize well-designed, prospective, bone metastasis-specific clinical trials to determine whether these mechanistic insights can translate into meaningful survival benefits and improved quality of life for patients with advanced bone metastases.

## Data Availability

Not applicable. This review article does not involve the generation or analysis of new experimental data.

## Abbreviations

ADAM10/17	a disintegrin and metalloproteinase domain-containing protein 10/17
AhR	aryl hydrocarbon receptor
ARG-1/2	arginase 1/2
BMAs	bone marrow adipocytes
CAR-T	chimeric antigen receptor T cell
CCL2	C-C motif chemokine ligand 2
CD8^+^ T cell	CD8-positive cytotoxic T lymphocyte
CTCs	circulating tumor cells
CTLs	cytotoxic T lymphocytes
CXCL12	C-X-C motif chemokine ligand 12
DCs	dendritic cells
DKK1	dickkopf WNT signaling pathway inhibitor 1
DNMT3A	DNA methyltransferase 3A
ECM	extracellular matrix
EVs	extracellular vesicles
FAO	fatty-acid oxidation
GLS	glutaminase
GLUT1	glucose transporter 1
GM-CSF	granulocyte-macrophage colony-stimulating factor
HK2	hexokinase 2
ICI(s)	immune checkpoint inhibitor(s)
iNOS	inducible nitric oxide synthase
IDO	indoleamine 2,3-dioxygenase
IFN-γ	interferon-gamma
IL	interleukin
irAEs	immune-related adverse events
LAG-3	lymphocyte activation gene-3
LDs	lipid droplets
lncRNA	long non-coding RNA
M-MDSCs	monocytic myeloid-derived suppressor cells
MCT1/4	monocarboxylate transporters 1 and 4
MDSCs	myeloid-derived suppressor cells
MICA/B	MHC class I polypeptide-related sequence A/B
miRNA	microRNA
MMP-9	matrix metalloproteinase-9
MRD	minimal residual disease
NF-κB	nuclear factor-kappa B
NKG2D	natural killer group 2 member D
NK cells	natural killer cells
ONJ	osteonecrosis of the jaw
OPN	osteopontin
OXPHOS	oxidative phosphorylation
PD-1	programmed cell death protein 1
PD-L1	programmed death-ligand 1
PFKFB3	6-phosphofructo-2-kinase/fructose-2,6-biphosphatase 3
PMN	pre-metastatic niche
PMN-MDSCs	granulocytic/polymorphonuclear myeloid-derived suppressor cells
PFS	progression-free survival
PPP	pentose phosphate pathway
PTHrP	parathyroid hormone-related protein
RANKL	receptor activator of nuclear factor κB ligand
ROS	reactive oxygen species
SASP	senescence-associated secretory phenotype
SBRT	stereotactic body radiation therapy
SDF-1	stromal-cell-derived factor 1
Siglec	sialic-acid-binding immunoglobulin-like lectin
SREs	skeletal-related events
STAT3	signal transducer and activator of transcription 3
sEVs	small extracellular vesicles
TAMs	tumor-associated macrophages
TCA	tricarboxylic acid
TGF-β	transforming growth factor-beta
TIGIT	T-cell immunoreceptor with Ig and ITIM domains
TIM-3	T-cell immunoglobulin and mucin-domain containing-3
TMB	tumor mutation burden
TME	tumor microenvironment
Tpex	CD8^+^TCF1^+^ precursor exhausted T cells
Tregs	regulatory T cells
Bregs	regulatory B cells
TNF-α	tumor necrosis factor-alpha
VEGF	vascular endothelial growth factor
